# Case Report: Balloon aortic valvuloplasty with subsequent Impella support as bridge therapy to transcatheter aortic valve replacement in cardiogenic shock with severe aortic stenosis

**DOI:** 10.3389/fcvm.2025.1583801

**Published:** 2025-05-22

**Authors:** Yukihiro Watanabe, Jun Nakata, Hiroki Matsushita, Keita Saku, Kosuke Mozawa, Toshiki Seki, Yukichi Tokita, Yuki Izumi, Masayuki Tsutsumi, Yu Hoshika, Tokuhiro Kimura, Masaaki Hino, Reiko Shiomura, Hideto Sangen, Takeshi Yamamoto, Kuniya Asai

**Affiliations:** ^1^ Division of Cardiovascular Intensive Care, Nippon Medical School Hospital, Tokyo, Japan; ^2^ Department of Cardiovascular Dynamics, National Cerebral and Cardiovascular Center Research Institute, Osaka, Japan; ^3^ Department of Cardiovascular Medicine, Nippon Medical School, Tokyo, Japan

**Keywords:** severe aortic stenosis, cardiogenic shock, balloon aortic valvuloplasty, impella, transcatheter aortic valve replacement, BAV-PELLA-TAVR

## Abstract

**Introduction:**

Cardiogenic shock (CS) with severe aortic stenosis (AS) is a drug-resistant hemodynamically unstable condition with high mortality. We report three cases of CS with severe AS that were successfully managed with balloon aortic valvuloplasty (BAV), followed by left ventricular (LV) unloading using Impella as a bridge therapy for transcatheter aortic valve replacement (TAVR). We call this therapeutic approach “BAV-PELLA-TAVR”.

**Case presentation:**

Case 1: A 92-year-old Japanese female presented with CS due to low-flow, low-gradient severe AS and multivessel coronary artery disease. After emergent BAV and Impella 2.5 support, the patient's hemodynamics stabilized. Percutaneous coronary intervention was performed on the right coronary and left anterior descending arteries with Impella 2.5 support. Subsequently, her heart failure (HF) improved and elective TAVR was performed. Case 2: An 89-year-old Japanese female presented with CS due to severe AS. Despite administration of high-dose catecholamines, the patient developed exacerbation of CS due to reduced cardiac output, corresponding to Stage D according to the Society for Cardiovascular Angiography and Interventions (SCAI) classification. Consequently, BAV was performed, which reduced the aortic valve pressure gradient (PG). However, due to persistent hemodynamic instability, Impella 2.5 support was initiated. This procedure resulted in hemodynamic improvement and elective TAVR was performed. Case 3: An 86-year-old Japanese female developed CS with pulmonary edema due to severe AS. Emergent BAV was performed. However, there was no improvement in the PG and hemodynamics, and the initial mild aortic regurgitation worsened to a moderate degree. Therefore, an Impella CP was implanted, which resulted in improved hemodynamics. Following the removal of the Impella CP device, and sub-emergent TAVR was successfully performed.

**Discussion:**

In all cases, emergent BAV and subsequent hemodynamic support from the Impella were provided as the initial treatment for CS at Stage C/D according to the SCAI classification. This approach improved CS, enabling interventions for concomitant ischemic heart disease, multidisciplinary heart team evaluation, and TAVR with reduced perioperative risk.

## 1 Introduction

Cardiogenic shock (CS) is a life-threatening condition characterized by tissue hypoperfusion due to low cardiac output (CO) (1). CS is caused by diseases involving impaired function of the myocardium, valve, conduction system, or pericardium, either in isolation or in combination (2). The incidence of aortic stenosis (AS) has been increasing worldwide with an aging population (3), and it is one of the major causes of CS and acute decompensated heart failure (ADHF). Furthermore, CS with severe AS is associated with high mortality and morbidity (4).

In recent years, transcatheter aortic valve replacement (TAVR) has become an established standard treatment for high-risk patients with severe AS (5, 6). However, in the setting of CS, TAVR is generally not indicated due to uncertain benefits and a high risk of complications. In a multicenter retrospective observational study, patients with decompensated AS who underwent emergency TAVR had poor outcomes, with a 30-day mortality of 24% and a high incidence of complications (vascular complications: 22%; stroke: 9%). Meanwhile, a staged treatment approach involving emergency balloon aortic valvuloplasty (BAV) followed by elective TAVR has also been widely adopted; however, this strategy similarly resulted in poor outcomes (7). Therefore, current therapeutic options have limited effectiveness, highlighting the need to establish effective treatment strategies for this condition.

We successfully performed BAV with subsequent hemodynamic support using Impella (Abiomed, Danvers, MA) as bridge therapy for TAVR. Several recent reports have supported this approach as a feasible and effective therapeutic option (8–11). We call this approach “BAV-PELLA-TAVR” and propose it as the optimal therapeutic strategy for CS with severe AS. The combination of aortic valve pressure gradient (PG) reduction by BAV and left ventricular (LV) unloading with Impella could stabilize hemodynamics and enable TAVR with reduced perioperative risk. This report presents three cases treated with BAV-PELLA-TAVR and discusses the hemodynamic advantages of this therapeutic approach.

## 2 Case presentation

### 2.1 Case 1: Low flow, low gradient (LF-LG) severe AS with multivessel coronary artery disease

**Figure 1:**
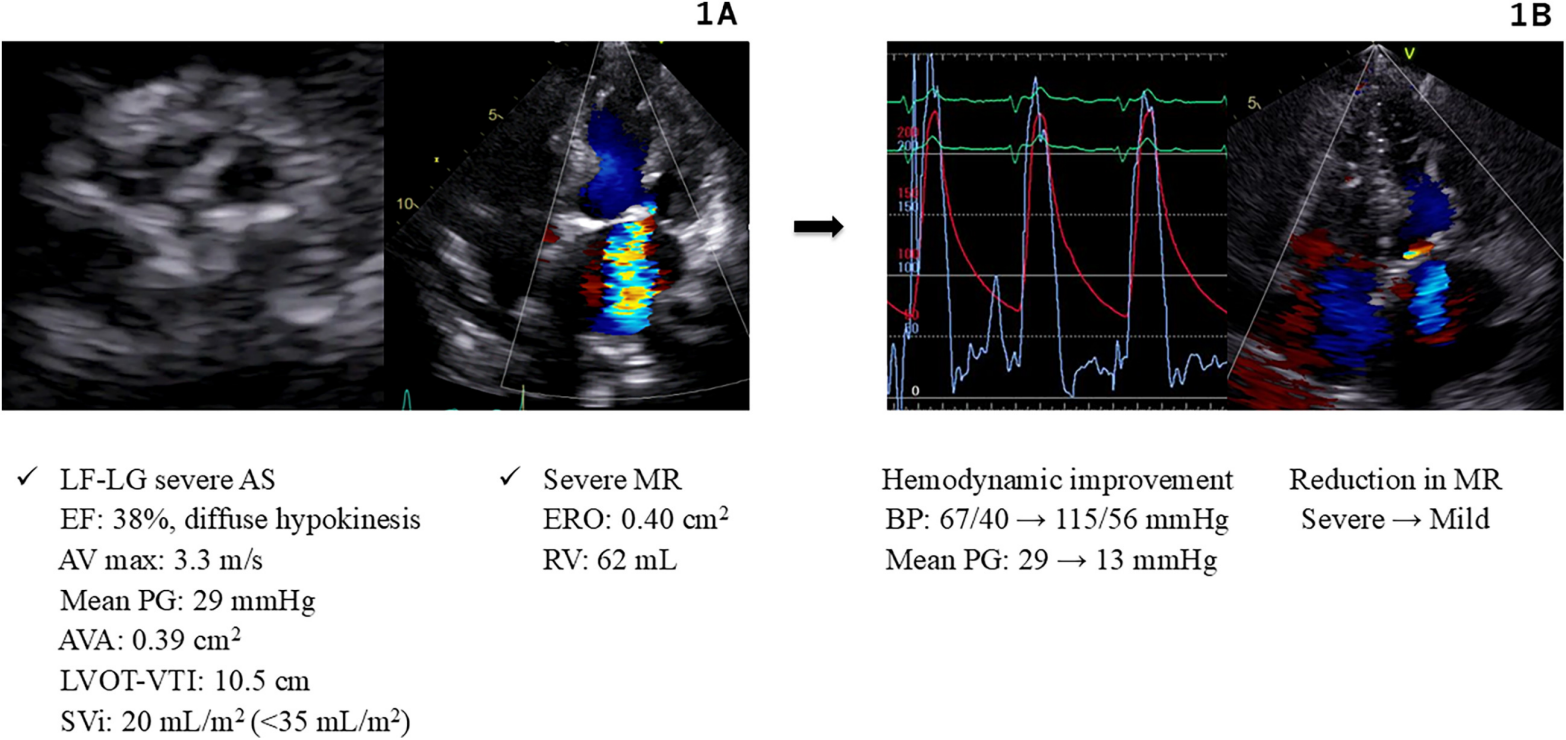
Hemodynamics and images in Case 1. **(A)** Echocardiography on admission (Case 1). **(B)** Hemodynamic improvement after BAV and Impella support (Case 1). AS, aortic stenosis; AV, aortic valve; AVA, aortic valve area; BAV, balloon aortic valvuloplasty; BP, blood pressure; EF, ejection fraction; ERO, effective regurgitant orifice; LF-LG, low flow low gradient; LVOT-VTI, left ventricular outflow tract velocity time integral; MR, mitral regurgitation; PG, pressure gradient; RV, regurgitant volume; SVi, stroke volume index.

A 92-year-old Japanese female with a medical history of hypertension and diabetes was admitted to our cardiovascular intensive care unit for ADHF. On admission, her vital signs were as follows: blood pressure, 103/65 mmHg; heart rate, 160 beats/min; and peripheral oxygen saturation, 95%, while receiving supplemental oxygen at 10 L/min. Blood tests showed an elevated high-sensitivity troponin T level of 0.243 ng/ml. Chest radiography revealed a cardiothoracic ratio of 66% and pulmonary congestion. Electrocardiography (ECG) showed atrial fibrillation with a heart rate of 150 beats/min and poor R-wave progression. Echocardiography revealed diffuse hypokinesis with a reduced left ventricular ejection fraction (LVEF) of 38%, aortic valve calcification, and severe mitral regurgitation (MR). The aortic valve peak velocity was 3.3 m/s, and the mean PG was 29 mmHg; however, the aortic valve area (AVA) was markedly low at 0.39 cm^2^ (calculated using the continuity equation), and the stroke volume index was also low at 20 ml/m^2^, suggesting LF-LG severe AS (Figure 1A).

Non-invasive positive pressure ventilation and intravenous furosemide administration were initiated. Landiolol was administered to manage tachycardic atrial fibrillation; however, as rate control remained inadequate, electrical cardioversion was performed, which successfully restored the sinus rhythm. Despite these initial treatments, the patient's blood pressure decreased to 77/37 mmHg, and the serum lactate level was elevated to 2.2 mmol/L, indicating Stage C according to the Society for Cardiovascular Angiography and Interventions classification (SCAI). Subsequently, mechanical ventilation was initiated, and norepinephrine at 0.3 γ and dobutamine at 3 γ were administered, resulting in an increase in blood pressure to 99/50 mmHg. Cardiac catheterization was performed to evaluate hemodynamics and coronary arteries. Right heart catheterization demonstrated the following hemodynamic parameters: pulmonary capillary wedge pressure (PCWP) of 23 mmHg, pulmonary artery pressure (PAP) of 50/20 mmHg, central venous pressure (CVP) of 13 mmHg, CO of 2.7 L/min (measured by the Fick method), and cardiac index (CI) of 1.9 L/min/m^2^. Coronary angiography (CAG) revealed multivessel coronary artery disease with 90% stenosis in the proximal and mid-right coronary artery, 90% stenosis in the proximal and mid-left anterior descending artery, and 75% stenosis in the proximal and mid-circumflex artery.

The cause of CS was considered to be non-ST-elevation myocardial infarction associated with LF-LG severe AS, severe MR, and ischemic heart disease. Consequently, we planned to improve hemodynamics through an intervention for severe AS by performing BAV using an 18-mm Tyshak balloon (Cardinal Health Japan, Tokyo, Japan). Immediately after the procedure, the patient experienced cardiac arrest owing to pulseless electrical activity. Cardiopulmonary resuscitation was promptly initiated, and an Impella 2.5 device was implanted, resulting in the stabilization of blood pressure at 115/56 mmHg. These procedures led to a reduction in the aortic valve PG from 29 to 13 mmHg, and the MR improved significantly (Figure 1B). Subsequently, percutaneous coronary intervention (PCI) was performed and drug-eluting stents (Xience Alpine; Abbott Vascular, Santa Clara, CA) were implanted into the right coronary artery and left anterior descending artery. These interventions stabilized the patient's hemodynamics, allowing discontinuation of catecholamines. The Impella device was removed on day 5, and the patient was extubated on day 9. Thereafter, the patient maintained compensated heart failure (HF) and stable hemodynamics with oral therapy alone. The heart team evaluation deemed the patient to be at a high surgical risk (STS score: 12.8%, Clinical Frailty Scale score: 4, Katz Index: 4), and TAVR was recommended. Elective TAVR with a 23-mm SAPIEN 3 valve (Edwards Lifesciences, Irvine, CA) was successfully performed on day 19.

### 2.2 Case 2: reduced cardiac output due to severe AS

An 89-year-old Japanese female with a history of hypertension and dyslipidemia was admitted to another hospital for ADHF. Owing to worsening hemodynamics, she was transferred to our hospital for advanced intensive care. On admission, the patient was intubated and on mechanical ventilation, with continuous infusions of norepinephrine at 0.3 γ and dopamine at 4 γ. Her vital signs were as follows: blood pressure, 94/61 mmHg; heart rate, 100 beats/min; and peripheral oxygen saturation, 100%, while receiving an FiO2 of 0.4. Chest radiography revealed pleural effusion, slight pulmonary congestion and a cardiothoracic ratio of 62%. ECG revealed atrial fibrillation with a heart rate of 129 beats/min and ST-segment depression in leads V4-V6. Echocardiography demonstrated a reduced LVEF of 37% and severe AS, with an aortic valve peak velocity of 5.8 m/s, a mean PG of 82 mmHg, and an AVA of 0.31 cm^2^. In addition, a septal e' velocity of 3.2 cm/s, an E/e’ ratio of 23.8, and a left atrial volume index of 113 ml/m^2^ were suggestive of LV diastolic dysfunction.

Electrical cardioversion was performed to manage atrial fibrillation and successfully restore the sinus rhythm. Despite the administration of high-dose catecholamines, hemodynamics remained unstable, and the serum lactate level was elevated at 2.4 mmol/L, indicating SCAI SHOCK Stage D. Right heart catheterization demonstrated the following hemodynamic parameters: PCWP of 10 mmHg, PAP of 25/12 mmHg, CVP of 5 mmHg, CO of 1.6 L/min (measured by the Fick method), and CI of 1.2 L/min/m^2^. CAG revealed no significant stenosis.

**Figure 2:**
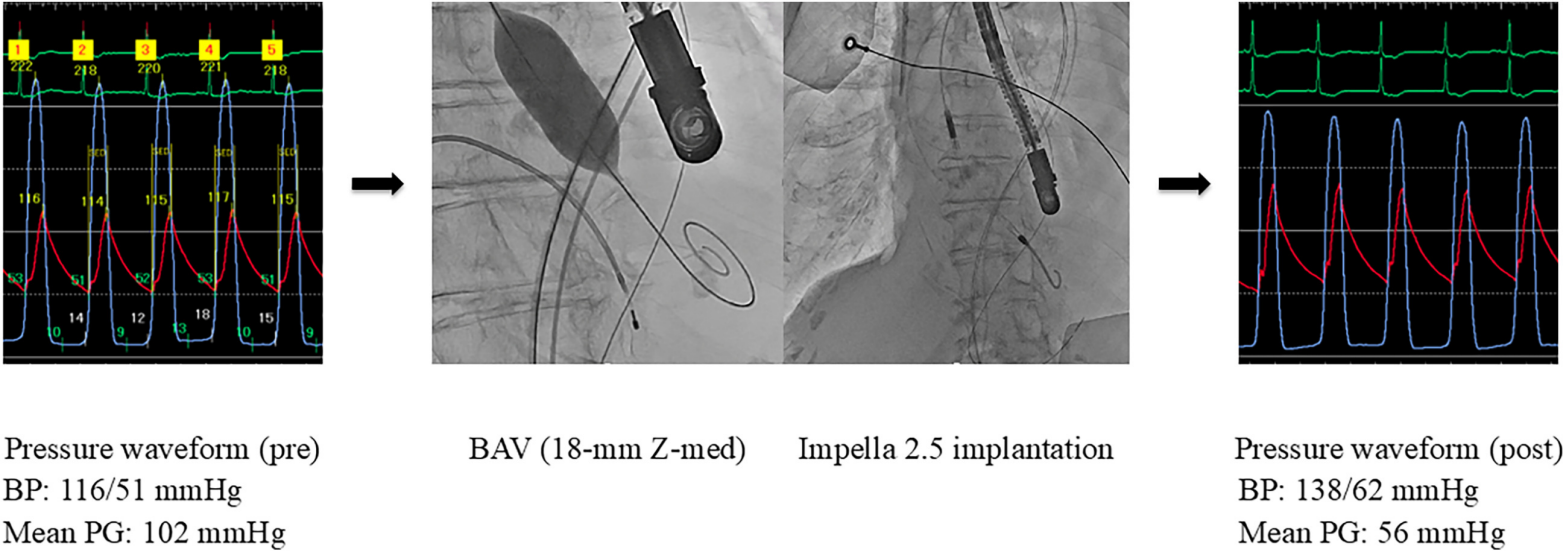
Hemodynamic improvement after BAV and Impella support (Case 2). BAV, balloon aortic valvuloplasty; BP, blood pressure; PG, pressure gradient.

The pathology was considered CS associated with reduced cardiac output due to severe AS. Emergent BAV was performed using an 18-mm Z-Med balloon (NuMED, Inc., Hopkinton, NY), resulting in a reduction in the aortic valve PG from 102 to 56 mmHg. However, low cardiac output persisted with a CO of 1.8 L/min and a CI of 1.4 L/min/m^2^. Subsequently, an Impella 2.5 device was implanted, selected because of poor vascular access and small body surface area of 1.2 m^2^. These interventions resulted in an increase in CO to 2.3 L/min, CI to 1.7 L/min/m^2^, and blood pressure to 138/62 mmHg (Figure 2). As the patient's cardiac function showed gradual improvement and hemodynamic parameters stabilized, the Impella device was removed on day 6. In the multidisciplinary heart team discussion, it was determined that the patient was at high surgical risk (STS score: 32.2%, Clinical Frailty Scale score: 2, Katz Index: 5), and TAVR was planned. On day 9, elective TAVR with a 23-mm SAPIEN 3 valve was successfully performed.

### 2.3 Case 3: cardiogenic pulmonary edema due to severe AS

An 86-year-old Japanese female with a history of hypertension was admitted to our cardiovascular intensive care unit for ADHF. On admission, her vital signs were as follows: blood pressure, 165/87 mmHg; heart rate, 85 beats/min; and peripheral oxygen saturation, 97%, while receiving 6 L/min of supplemental oxygen. Chest radiography revealed a cardiothoracic ratio of 60%, pulmonary congestion, and pleural effusion. ECG showed sinus rhythm with a heart rate of 88 beats/min and ST-segment depression in leads V4-V6. An echocardiogram demonstrated a LVEF of 55%, mild aortic regurgitation (AR), and severe AS, with an aortic valve peak velocity of 4.7 m/s, a mean PG of 63 mmHg, and an AVA of 0.21 cm^2^.

After initiating mechanical ventilation, the blood pressure decreased, and the serum lactate level was elevated at 2.0 mmol/L, indicating SCAI SHOCK Stage C. Administration of norepinephrine at 0.1 γ was initiated immediately. Right heart catheterization demonstrated the following hemodynamic parameters: PCWP of 42 mmHg, PAP of 51/37 mmHg, CVP of 17 mmHg, CO of 3.7 L/min (measured by the Fick method), and CI of 2.6 L/min/m^2^. CAG showed no significant stenosis. The peak-to-peak gradient between the left ventricle and aorta (LV-Ao) was 100 mmHg.

**Figure 3:**
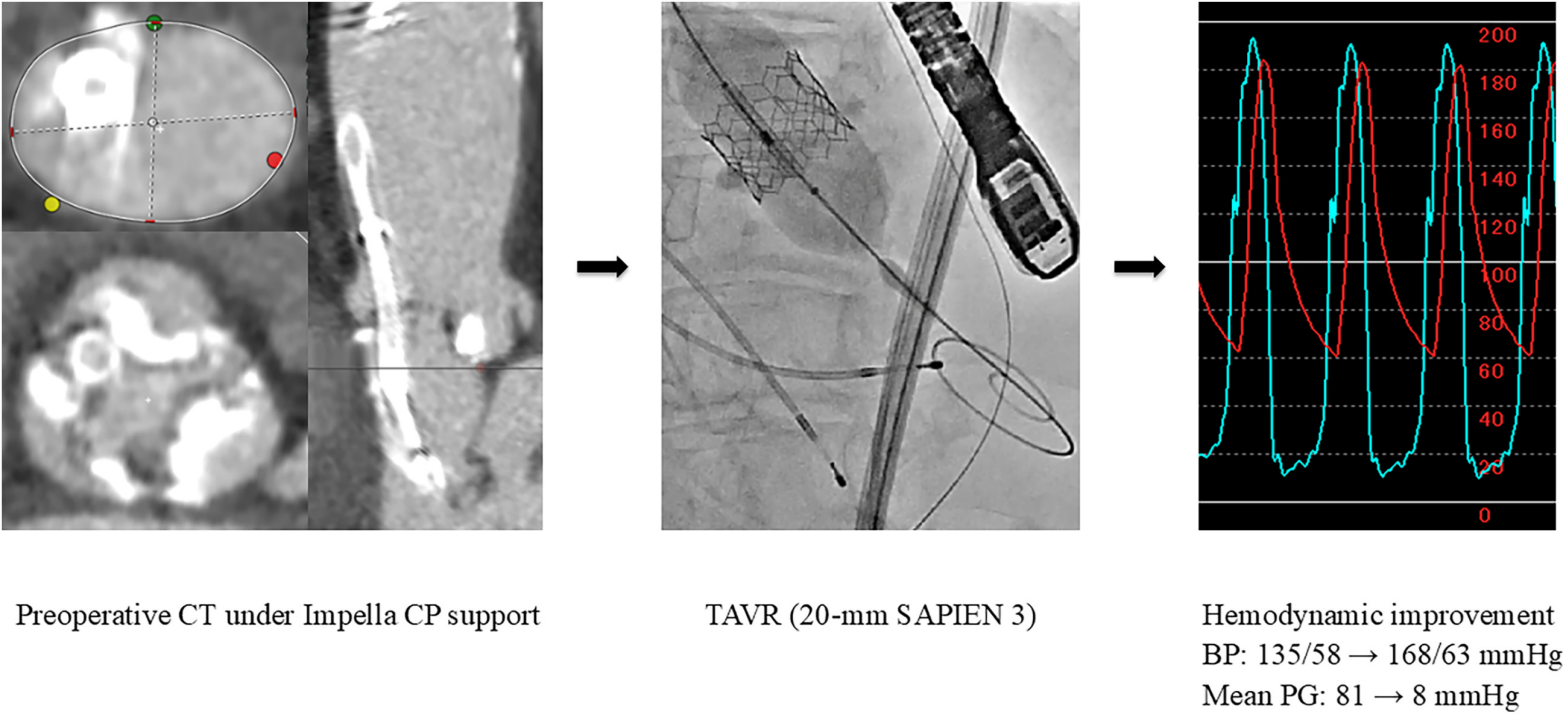
Preoperative CT imaging and urgent TAVR (Case 3). BP, blood pressure; CT, computed tomography; PG, pressure gradient; TAVR, transcatheter aortic valve replacement.

The pathology was CS with pulmonary edema caused by severe AS. Emergent BAV was performed using a 16-mm Z-Med balloon. However, the LV-Ao PG remained high at 80 mmHg, and the PCWP was high at 42 mmHg. Furthermore, the severity of AR worsened from mild to moderate; thus, an additional oversized BAV was determined to carry a high risk of further AR exacerbation. Consequently, an Impella CP device was implanted, which led to an increase in the blood pressure, allowing for the discontinuation of catecholamines, and a reduction in PCWP to 15 mmHg. The heart team evaluation determined that the patient was at a high surgical risk (STS score: 22.2%, Clinical Frailty Scale score: 4, Katz Index: 4), and TAVR was planned. Preoperative computed tomography performed under Impella CP support demonstrated the following measurements: an aortic valve annulus with diameters of 16.3 × 23.3 mm, a perimeter of 63.2 mm, and an area of 303 mm^2^. On day 7, following the removal of the Impella CP device, sub-emergent TAVR with a 20-mm SAPIEN 3 valve was successfully performed, reducing the aortic valve PG from 81 to 8 mmHg (Figure 3). Subsequently, adequate diuresis was achieved, pulmonary congestion improved, and the patient was extubated on day 9. HF remained compensated with oral medications alone. Cardiac rehabilitation was carried out, resulting in improved activities of daily living, and the patient was discharged home on day 28.

## 3 Discussion

Patients with severe AS often develop ADHF and CS, resulting in poor prognosis (12, 13). Several case series have demonstrated the value of BAV in achieving hemodynamic stability before definitive therapy (14, 15). However, BAV has intrinsic risks, and its efficacy is modest, with a risk of significant AR.

On the other hand, emergent or urgent TAVR can be an optional therapy (16). Classically, patients with CS are not considered candidates for TAVR because of concerns regarding the feasibility of a standard pre-procedural evaluation including computed tomography and the consequent risks of inaccurate transcatheter aortic valve sizing, improper assessment of periprocedural complications, and unsuitability of iliofemoral access, as well as the risk of complications associated with the procedure itself (7). Nonetheless, in light of the results of a substudy of the Society of Thoracic Surgeons and the American College of Cardiology Transcatheter Valve Therapy registry, “primary” TAVR has demonstrated a high procedural success rate in patients with CS (17). Despite this, the 30-day mortality rate remains high at 19.1%, suggesting that primary TAVR alone may not lead to sufficient improvement in clinical outcomes. In contrast, BAV, as a bridge to definitive therapy, may be considered in hemodynamically unstable patients who are at high risk for surgery (5, 15). Thus, emergent BAV with subsequent hemodynamic support by mechanical circulatory support, followed by elective or urgent TAVR after hemodynamic stabilization, is essentially an ideal approach for CS with severe AS.

According to the Japanese Circulation Society (JCS)/Japanese Society for Cardiovascular Surgery (JSCVS)/Japanese College of Cardiology (JCC)/Japanese Association of Cardiovascular Intervention and Therapeutics (CVIT) 2023 guidelines focused on the indication and operation of percutaneous cardiopulmonary support (PCPS)/extracorporeal membrane oxygenation (ECMO)/Impella, intravenous inotropic drugs are recommended to be administered first for patients with SCAI SHOCK Stages C and D. Coronary revascularization, such as PCI, should be performed if necessary, and Impella should be used if hypoperfusion persists, accompanied by elevated left ventricular end-diastolic pressure (LVEDP) (18). Physiologically, patients with severe AS are not eligible for Impella because the insertion of the device through the stenotic valve orifice is technically challenging. However, emergent BAV with a small balloon to achieve the minimum diameter makes it possible to insert an Impella device. We measured the aortic annulus diameter using transesophageal echocardiography prior to BAV and selected a balloon size that did not exceed the measured value. This allowed for successful Impella insertion across the aortic valve in all cases. Thus, “BAV-PELLA,” which refers to BAV and hemodynamic support with Impella, may be an ideal option as a reasonable and safe intervention for CS caused by severe AS.

**Figure 4:**
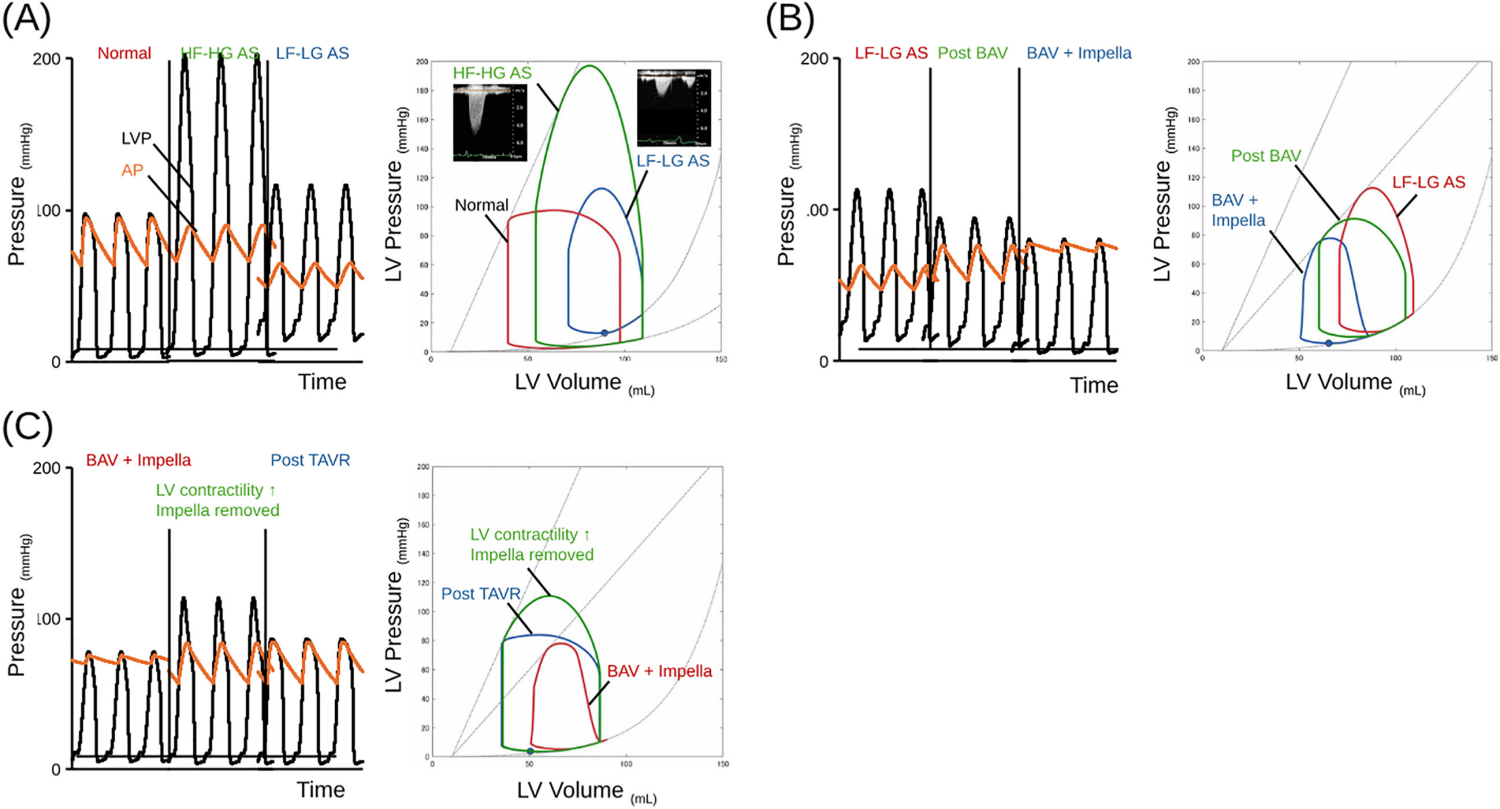
The schema of trends in hemodynamics and PV loop in LF-LG AS case treating BAV-PELLA-TAVR. **(A)** Hemodynamics and PV loops of a normal heart, AS, and LF-LG AS patients. The schema of hemodynamics and PV loops were illustrating by hemodynamic simulator. The AS causes a significant upward shift in the PV loop (green line) from the baseline (red line). Impaired LV systolic and diastolic function shifts the PV loop downward (blue line). The details of the simulation model can be found in reference (17). In the simulation, each ventricular valve was modeled as a one-way valve based on Bernoulli's theorem, while AS was simulated by varying the AVA from 4.0 to 0.4 cm^2^/s. **(B)** Hemodynamics and PV loops of BAV in an LF-LG AS patient with low LV function followed by Impella support. BAV significantly reduces the PG, while the increase of stroke volume is limited, and congestive HF is not sufficiently improved (green line). Impella significantly improves hemodynamics while reducing the LV load (blue line). **(C)** Hemodynamics and PV loops in a LF-LG AS patient transitioning to TAVR. Impella support after BAV provides stable hemodynamics, contributes to improved LV contractility (green line), and allows TAVR (blue line) to be performed in a stable condition. AP, aortic pressure; AS, aortic stenosis; BAV, balloon aortic valvuloplasty; AVA, aortic valve area; HF, heart failure; HF-HG, high flow high gradient; LF-LG, low flow low gradient; LV, left ventricular; LVP, left ventricular pressure; PG, pressure gradient; PV loop, pressure-volume loop; TAVR, transcatheter aortic valve replacement.

The treatment sequence of BAV-PELLA-TAVR is recommended for AS patients with impaired cardiac function in whom congestion and low CO are likely to persist even after BAV. Figure 4 illustrates the hemodynamics of LF-LG AS with impaired LV function and the treatment process of BAV-PELLA-TAVR using our previously developed cardiovascular simulator (19, 20). Figure 4A shows the hemodynamics and pressure-volume (PV) loops of a normal heart, AS, and LF-LG AS. AS causes a significant increase in the PG and an upward shift in the PV loop, suggesting an increase in LV mechanical work. However, in patients with normal LV function, changes in CO, atrial pressure, and left ventricular end-diastolic volume (LVEDV) remain within acceptable ranges without major circulatory collapse. In contrast, with impaired LV systolic and diastolic functions, the PV loop shifts downward and the PG also significantly decreases. Figure 4B shows BAV in a LF-LG AS patient with low LV function. Although BAV significantly reduces PG, the resulting increase in stroke volume is limited, and congestive HF may not be sufficiently improved. Using Impella in such cases can significantly improve hemodynamics while reducing the LV load. In some cases, Impella-mediated HF management can even improve LV function. As shown in Figure 4C transitioning to TAVR with well-managed HF enables the safe radical treatment of AS.

Case 1 most closely resembles the simulation shown in Figure 4. Impella-mediated HF management and PCI may contribute to improved LV function. In Case 2, cardioversion was promptly performed for atrial fibrillation after transfer, successfully restoring sinus rhythm. The recovery of atrial function can increase the inflow from the left atrium to the LV. However, in this case with severe AS and LV diastolic dysfunction, the increased LV inflow due to sinus rhythm restoration may not have contributed to an increase in CO and could have exacerbated LV load. Although severe AS may have contributed to the reduced CO, the use of vasopressors and diuretics for congestion relief and blood pressure maintenance might have caused a significant preload reduction and afterload increase, potentially leading to a persistently low CO after BAV. Impella support promptly improved hemodynamics and enabled stable medication adjustment and pre-TAVR HF management. Case 3 had severe AS with preserved LV function. As BAV treatment was limited owing to worsening AR, Impella was used for HF management, enabling a stable transition to TAVR. Despite differences in LV function, AS severity, and HF status, all three cases shared the common feature that Impella-mediated HF management after BAV facilitated a stable transition to TAVR treatment. In addition, BAV-PELLA-TAVR provided sufficient time to discuss the optimal strategy for severe AS in the heart team meeting.

## 4 Conclusions

“BAV-PELLA-TAVR,” which stabilizes hemodynamics and improves ADHF, may be one of the optimal therapeutic options for the sickest patients with CS due to severe AS.

## Data Availability

The datasets presented in this article are not readily available because the raw data supporting the conclusions of this article are not publicly available due to concerns regarding patient confidentiality. Relevant information has been anonymized and summarized in the article. Further details may be provided upon reasonable request, contingent on approval by the institutional ethics board. Requests to access the datasets should be directed to Jun Nakata, jun-nakata@nms.ac.jp.

## References

[1] Lüsebrink E Binzenhöfer L Adamo M Lorusso R Mebazaa A Morrow DA Cardiogenic shock. Lancet. (2024) 404:2006–20. 10.1016/s0140-6736(24)01818-x 39550175

[2] Nakata J Yamamoto T Saku K Ikeda Y Unoki T Asai K. Mechanical circulatory support in cardiogenic shock. J Intensive Care. (2023) 11:64. 10.1186/s40560-023-00710-2 38115065 PMC10731894

[3] d'Arcy JL Coffey S Loudon MA Kennedy A Pearson-Stuttard J Birks J Large-scale community echocardiographic screening reveals a major burden of undiagnosed valvular heart disease in older people: the OxVALVE population cohort study. Eur Heart J. (2016) 37:3515–22. 10.1093/eurheartj/ehw229 27354049 PMC5216199

[4] Nair RM Chawla S Abdelghaffar B Alkhalaieh F Bansal A Puri R Comparison of contemporary treatment strategies in patients with cardiogenic shock due to severe aortic stenosis. J Am Heart Assoc. (2024) 13:e033601. 10.1161/jaha.123.033601 38761069 PMC11179830

[5] Otto CM Nishimura RA Bonow RO Carabello BA Erwin JP 3rd Gentile F 2020 ACC/AHA guideline for the management of patients with valvular heart disease: a report of the American College of Cardiology/American Heart Association Joint Committee on clinical practice guidelines. Circulation. (2021) 143:e72–e227. 10.1161/cir.0000000000000923 33332150

[6] Izumi C Eishi K Ashihara K Arita T Otsuji Y Kunihara T JCS/JSCS/JATS/JSVS 2020 guidelines on the management of valvular heart disease. Circ J. (2020) 84:2037–119. 10.1253/circj.CJ-20-0135 32921646

[7] Bongiovanni D Kühl C Bleiziffer S Stecher L Poch F Greif M Emergency treatment of decompensated aortic stenosis. Heart. (2018) 104:23–9. 10.1136/heartjnl-2016-311037 28566471

[8] Johnson DW Erwin IJ. Use of Impella 5.0 prior to transcatheter aortic valve replacement in a patient with severe aortic stenosis and cardiogenic shock. J Heart Valve Dis. (2017) 26:485–7. 29302950

[9] Burzotta F Nerla R Trani C. Bail-ouse of impella CP as a bridge to TAVI in a cardiogenic shock patient: the “pump-rewiring” technique. J Invasive Cardiol. (2016) 28:E1–5. 26716594

[10] Panoulas V Greenough N Sulemane S Monteagudo-Vela M Lees N. The role of mechanical circulatory support in patients with severe left ventricular impairment treated with transcatheter aortic valve implantation and percutaneous coronary intervention. Cardiovasc Revasc Med. (2021) 28s:169–75. 10.1016/j.carrev.2021.03.020 33875387

[11] Abraham J Wang L Kumar V Kirker EB Spinelli KJ. Axillary transvalvular microaxial pump as extended bridge to transcatheter aortic valve replacement in cardiogenic shock with severe aortic stenosis. J Heart Lung Transplant. (2022) 41:434–7. 10.1016/j.healun.2021.12.010 35090810

[12] Miura S Arita T Kumamaru H Domei T Yamaji K Soga Y Causes of death and mortality and evaluation of prognostic factors in patients with severe aortic stenosis in an aging society. J Cardiol. (2015) 65:353–9. 10.1016/j.jjcc.2015.02.011 25890579

[13] Goel K Shah P Jones BM Korngold E Bhardwaj A Kar B Outcomes of transcatheter aortic valve replacement in patients with cardiogenic shock. Eur Heart J. (2023) 44:3181–95. 10.1093/eurheartj/ehad387 37350747 PMC10471523

[14] Eugène M Urena M Abtan J Carrasco JL Ghodbane W Nataf P Effectiveness of rescue percutaneous balloon aortic valvuloplasty in patients with severe aortic stenosis and acute heart failure. Am J Cardiol. (2018) 121:746–50. 10.1016/j.amjcard.2017.11.048 29397882

[15] Debry N Kone P Vincent F Lemesle G Delhaye C Schurtz G Urgent balloon aortic valvuloplasty in patients with cardiogenic shock related to severe aortic stenosis: time matters. EuroIntervention. (2018) 14:e519–e25. 10.4244/eij-d-18-00029 29741481

[16] Kitahara H Kumamaru H Kohsaka S Yamashita D Kanda T Matsuura K Clinical outcomes of urgent or emergency transcatheter aortic valve implantation- insights from the nationwide registry of Japan transcatheter valve therapies. Circ J. (2024) 88:439–47. 10.1253/circj.CJ-22-0536 36575039

[17] Masha L Vemulapalli S Manandhar P Balan P Shah P Kosinski AS Demographics, procedural characteristics, and clinical outcomes when cardiogenic shock precedes TAVR in the United States. JACC Cardiovasc Interv. (2020) 13:1314–25. 10.1016/j.jcin.2020.02.033 32499022

[18] Nishimura T Hirata Y Ise T Iwano H Izutani H Kinugawa K JCS/JSCVS/JCC/CVIT 2023 guideline focused update on indication and operation of PCPS/ECMO/IMPELLA. J Cardiol. (2024) 84:208–38. 10.1016/j.jjcc.2024.04.006 39098794

[19] Hiraoka A Saku K Nishikawa T Sunagawa K. A case report of unexpected right-to-left shunt under mechanical support for post-infarction ventricular septal defect: evaluation with haemodynamic simulator. Eur Heart J Case Rep. (2021) 5:ytab209. 10.1093/ehjcr/ytab209 34514298 PMC8422329

[20] Yokota S Nishikawa T Saku K. Impella as an optimizing tool for heart failure interventions. J Coron Artery Dis. (2024) 30:127–37. 10.7793/jcad.30.23-00021

